# Gametocyte clearance dynamics following oral artesunate treatment of uncomplicated *falciparum* malaria in Malian children

**DOI:** 10.1051/parasite/2016003

**Published:** 2016-02-02

**Authors:** Abdoulaye A. Djimde, Amelia W. Maiga, Dinkorma Ouologuem, Bakary Fofana, Issaka Sagara, Demba Dembele, Sekou Toure, Kassim Sanogo, Souleymane Dama, Bakary Sidibe, Ogobara K. Doumbo

**Affiliations:** 1 Malaria Research and Training Center, Department of Epidemiology of Parasitic Diseases, Faculty of Pharmacy, University of Science, Techniques and Technologies of Bamako P.O. Box 1805 Mali; 2 Vanderbilt University Medical Center Nashville TN 37232 USA

**Keywords:** *Plasmodium falciparum*, Gametocyte clearance, Gametocyte density, Artesunate monotherapy

## Abstract

Artemisinin-based combination therapies decrease *Plasmodium* gametocyte carriage. However, the role of artesunate in monotherapy *in vivo*, the mechanisms involved, and the utility of gametocyte carriage as a potential tool for the surveillance of antimalarial resistance are poorly understood. In 2010–2011, we conducted an open-label, prospective efficacy study of artesunate as monotherapy in children 1–10 years of age with uncomplicated *falciparum* malaria in Bougoula-Hameau, Mali. Standard oral doses of artesunate were administered for 7 days and patients were followed up for 28 days. The data were compared to a similar study conducted in 2002–2004. Of 100 children enrolled in the 2010–2011 study, 92 were analyzed and compared to 217 children enrolled in the 2002–2004 study. The proportion of gametocyte carriers was unchanged at the end of treatment (23% at baseline vs. 24% on day 7, *p* = 1.0) and did not significantly decline until day 21 of follow-up (23% vs. 6%, *p* = 0.003). The mean gametocyte density at inclusion remained unchanged at the end of treatment (12 gametocytes/μL vs. 16 gametocytes/μL, *p* = 0.6). Overall, 46% of the 71 initial non-carriers had gametocytes detected by day 7. Similar results were found in the 2002–2004 study. In both studies, although gametocyte carriage significantly decreased by the end of the 28-day follow-up, artesunate did not clear mature gametocytes during treatment and did not prevent the appearance of new stage V gametocytes as assessed by light microscopy. Baseline gametocyte carriage was significantly higher 6 years after the deployment of artemisinin-based combination therapies in this setting.

## Abstract (fr)

Les combinaisons thérapeutiques à base d’artémisinine diminuent la prévalence de la gamétocytémie. Cependant, le rôle de l’artésunate en monothérapie, les mécanismes impliqués et l’utilité du portage de gamétocytes comme un outil potentiel pour la surveillance de la chimiorésistance à ces antipaludiques sont mal compris. En 2010-2011, nous avons mené une étude prospective ouverte de l’efficacité de l’artésunate en monothérapie chez des enfants âgés de 1 à 10 ans et atteints de paludisme simple à *Plasmodium falciparum* à Bougoula-Hameau, au Mali. Des doses standards d’artésunate ont été administrées *per os* et les patients ont été suivis pendant 28 jours. Les données ont été comparées à celles d’une étude similaire menée en 2002-2004. Sur les 100 enfants inscrits dans l’étude en 2010-2011, 92 ont été analysés et comparés à 217 enfants inclus dans l’étude de 2002-2004. La proportion de porteurs de gamétocytes était inchangée à la fin du traitement (23 % au départ contre 24 % au jour 7, *p* = 1.0) et n’a diminué de façon significative qu’au 21^e^ jour de suivi (23 % vs 6 %, *p* = 0.003). La densité (moyenne géométrique) des gamétocytes à l’inclusion est restée inchangée à la fin du traitement (12 gamétocytes/μL vs 16 gamétocytes/μL, *p* = 0.6). Globalement, 46 % des 71 patients non-porteurs initiaux avait des gamétocytes détectés entre le début du traitement et le jour 7. Des résultats similaires ont été obtenus lors de l’étude de 2002-2004. Lors de ces deux études, bien que le portage des gamétocytes ait considérablement diminué à la fin des 28 jours de suivi, l’artésunate n’avait ni éliminé les gamétocytes matures pendant le traitement ni empêché l’apparition de nouveaux gamétocytes de stade V, selon les observations de microscopie optique. Le taux de base de portage de gamétocytes était significativement plus élevé six années après l’introduction des combinaisons thérapeutiques à base d’artémisinine dans cette localité.

## Introduction

Although recent estimates suggest that malaria mortality rates decreased by an impressive 47% between 2000 and 2013 globally, and by 54% in the World Health Organization’s (WHO) African Region, malaria remains a major public health problem in a number of countries [46]. Malaria elimination and ultimate eradication will require drugs targeting all stages of the parasite’s life cycle. Yet, very few drugs are known to be effective on the sexual stages (gametocytes) of *Plasmodium falciparum*. Gametocyte formation within the host involves the differentiation of sexually committed merozoites that undergo five distinct developmental stages (stage I–V) over a period of ~10 days [4, 35, 39]. Immature gametocytes (stage I–III) are thought to be metabolically comparable to asexual blood stage parasites and likely to be sensitive to the same antimalarial drugs [21, 22]. In contrast, mature gametocytes (stage IV–V), which are the only stages circulating in the bloodstream, are less active metabolically and relatively insensitive to most drugs [21, 22]. Indeed, primaquine and methylene blue are the only antimalarials with proved efficacy *in vivo* against mature gametocytes [7]. Artemisinin-based combination therapies (ACTs) reduce *P. falciparum* gametocyte carriage [9, 43]. Specifically, artemisinins have been shown to have some early-stage gametocytocidal effects on both *in vitro* and in feeding experiments, which may lead to a reduction in malaria transmission [10, 21]. However, field studies showed that artesunate reduces but does not prevent post-treatment transmission of *P. falciparum* to *Anopheles gambiae* [10, 21, 27, 32, 40].

*Plasmodium falciparum* resistance to artemisinins has now been confirmed along the Thai-Cambodian border [1, 26], in the greater Mekong area [3], and is spreading [38, 42] as well as emerging *de novo* from different genetic and geographical backgrounds [37]. K13 propeller polymorphisms are associated with *in vitro* and *in vivo* artemisinin resistance in Asia and are proposed as a molecular marker of artemisinin resistance in the field [2]. No cases of malaria parasite resistance to artemisinin have been described in sub-Saharan Africa as yet [3, 23] but several non-synonymous polymorphisms of K13 propeller have been described [21, 43]. The high intensity of malaria transmission [28, 33] and high acquired antimalarial immunity in sub-Saharan Africa [13] may delay the manifestation or emergence of drug resistance in these settings as compared to South-East Asia. Therefore, alternative markers may be necessary for the monitoring of artemisinin resistance in sub-Saharan Africa. Gametocyte carriage and dynamics could provide a sensitive early warning sign of emerging drug resistance [7]. Reduced susceptibility to artesunate + mefloquine, as demonstrated by delayed parasite clearance, has been associated with an increased risk of gametocyte carriage in Thailand [9]. Previously, increased rates of gametocyte carriage predicted sulfadoxine-pyrimethamine resistance in South Africa before it was clinically apparent or detectable via measurable changes in parasite clearance [6, 44]. Presenting with a recrudescent infection is a known risk factor for the development or persistence of gametocytemia after treatment of uncomplicated *falciparum* malaria [31].

Here, we sought to investigate (1) whether artemisinin as monotherapy kills mature gametocytes *in vivo* and (2) whether gametocyte carriage could be used as a monitoring tool for the detection of early signs of artemisinin resistance in sub-Saharan Africa.

## Materials and methods

As described elsewhere [23], from December 2010 to March 2011 we conducted a one-armed prospective therapeutic efficacy study of seven-day curative artesunate monotherapy in 100 children from Bougoula-Hameau, Mali aged 1–10 years and presenting with uncomplicated *falciparum* malaria.

Inclusion and non-inclusion criteria have been previously described [14, 23]. Briefly, children were aged 1–10 years with uncomplicated malaria, defined as an axillary temperature of ≥ 37.5 °C or a history of fever in the past 24 h; a positive malaria blood smear with between 2000 and 200,000 *P. falciparum* trophozoites per microliter; and no signs of severe malaria as defined by WHO [47]. Mixed infections were included as long as the *P. falciparum* parasitemia fell within the inclusion range. Written informed consent was obtained from the parent or guardian of each child prior to inclusion. The Ethics Committee of the Faculty of Medicine, Pharmacy, and Odonto-Stomatology, University of Bamako approved both studies. Patients were followed up actively and passively for 28 days using a WHO standard protocol. Thick and thin blood smears were prepared and stained with 5% Giemsa and read for asexual and sexual parasite counts according to standard procedures. Smears were prepared immediately prior to treatment initiation, every 8 h thereafter until three consecutive slides were negative for asexual parasites, and then at 48 h, 72 h, and on follow-up days 7, 14, 21, and 28. Asexual parasite density was estimated as previously described [45]. Gametocyte density was calculated per 1000 leukocytes, assuming a mean of 8000 leukocytes/μL [45]. Expert double-read microscopy was used for quality control. Qualitative discrepancies were resolved by a third microscopist and quantitative discrepancies of > 50% were resolved by taking the average of a third microscopist’s result and that of whichever one of the two original results closest to the third. Artesunate as monotherapy (100 mg tablets, Asunate Denk^®^, Denk Pharma) was administered orally under the supervision of a designated clinician as single doses of 4 mg/kg on the first day and 2 mg/kg/day each day thereafter for a seven-day course. Although Asunate Denk^®^ is a loose combination of artesunate and sulfadoxine-pyrimethamine, only the artesunate tablets were used for this study. Doses were systematically rounded up to the nearest quarter tablet. A repeat full dose was re-administered in the case of rejection or vomiting in the first 30 min. Cases of treatment failure were treated according to Malian national guidelines.

Pre-specified outcomes in the 2010–2011 study included the 28-day per-protocol cure rate, the parasite clearance time (PCT), the gametocyte clearance time (GCT), and the fever clearance time (FCT). GCT was defined as the time from the first dose to the complete disappearance of gametocytes maintained for at least 48 h. For the purposes of this secondary analysis, we compared the gametocyte carriage and density at 0, 1, 2, 3, 7, 14, 21, and 28 days after treatment initiation. Gametocyte carriage was evaluated as a dichotomous present/absent at each time point. Gametocyte clearance time was estimated as a continuous variable for gametocyte carriers at the time of inclusion. Among non-carriers at the time of inclusion, the risk of developing gametocytemia during the follow-up period was calculated.

The study procedures closely paralleled those of the artesunate monotherapy arm of a randomized control trial conducted in 2002–2004 in the same village by the same study team [14]. The main differences from the 2010–2011 study were the following: (1) smears were prepared at the time of screening (day 0) and on follow-up days 1, 3, 7, 14, 21, and 28. For quality control purposes, a 10% sample of all slides was read by a second blinded microscopist; (2) artesunate as monotherapy (50 mg tablets, Arsumax^®^, Sanofi-Aventis) was administered as described above but for 5 days instead of 7 days. In each study, data were collected on case report forms and then double-entered into a Microsoft Access database. Categorical variables (e.g., gametocyte carriage) were compared using the chi-square test and the two-sided Fisher’s exact test. Continuous variables (e.g., gametocyte density) were compared using Student’s *t*-test or the Mann-Whitney test, as appropriate. All statistical analyses were done with Stata version 11.0 (StataCorp, College Station, TX, USA). *P* values less than 0.05 were considered statistically significant.

## Results

### Study population

Of the 100 children originally enrolled in the 2010–2011 study, 92 were included in this analysis. Six were excluded for having received an incorrect dose of artesunate on the first day of treatment, and two were excluded after their treatment was changed from artesunate to intramuscular artemether and quinine, respectively [23]. From the 2002–2004 study, a total of 217 children aged 1–10 years who received artesunate monotherapy were included in this analysis, of whom 76 were enrolled during the months of December and January.

### Dynamics of gametocyte carriage

**Figure 1.**
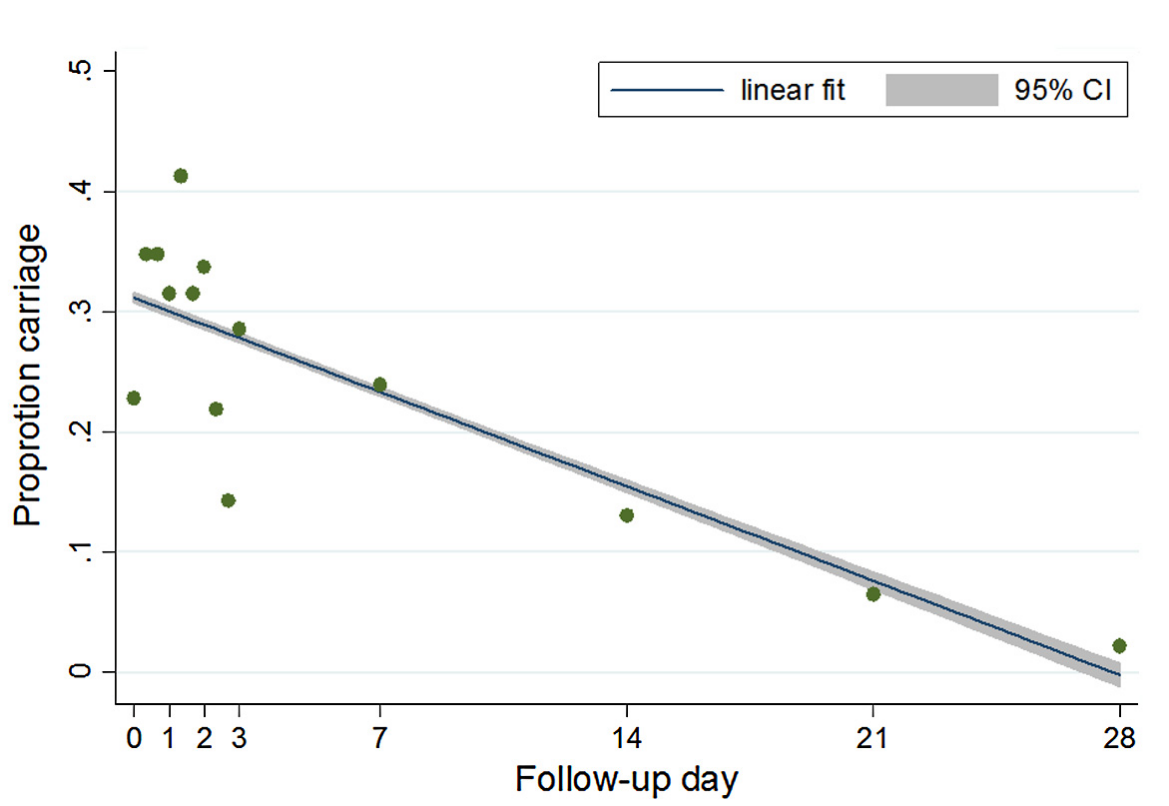
Evolution of gametocyte carriage: results are based on all children included from the 2010–2011 study (*n* = 92), not just initial carriers.

In the 2010–2011 study, 22.8% of children (21 of 92) were gametocyte carriers at the time of treatment initiation (Fig. 1). The proportion of gametocyte carriers was unchanged at the end of treatment on day 7 (23.9%, *n* = 92; *p* > 0.9). However, by day 21 of follow-up there was a significant decline of gametocyte carriage (6.5%, *n* = 92; *p* = 0.003). Of those initially gametocytemic, 20 (95.3%), 18 (85.7%), 16 (76.2%), 16 (76.2%), 9 (42.9%), 5 (23.8%), and 2 (9.5%) remained carriers at follow-up days 1, 2, 3, 7, 14, 21, and 28, respectively.

In the 2002–2004 study, 6% of children (13 of 217) were gametocyte carriers at the time of treatment initiation. The proportion of gametocyte carriers actually rose on day 1 of follow-up to 12.4% (*p* = 0.03), and did not drop significantly until day 21 of follow-up (0.5%, *p* = 0.002). Inter-study comparison shows that the gametocyte carriage rate was significantly higher in 2010–2011 than in 2002–2004 (22.8% vs. 6%, *p* < 0.0001). When the 2002–2004 study population was restricted to patients recruited during the months of December and January, the difference was still statistically significant (22.8%, *n* = 92 vs. 10.5%, *n* = 76; *p* = 0.04).

### Dynamics of gametocyte density

**Figure 2.**
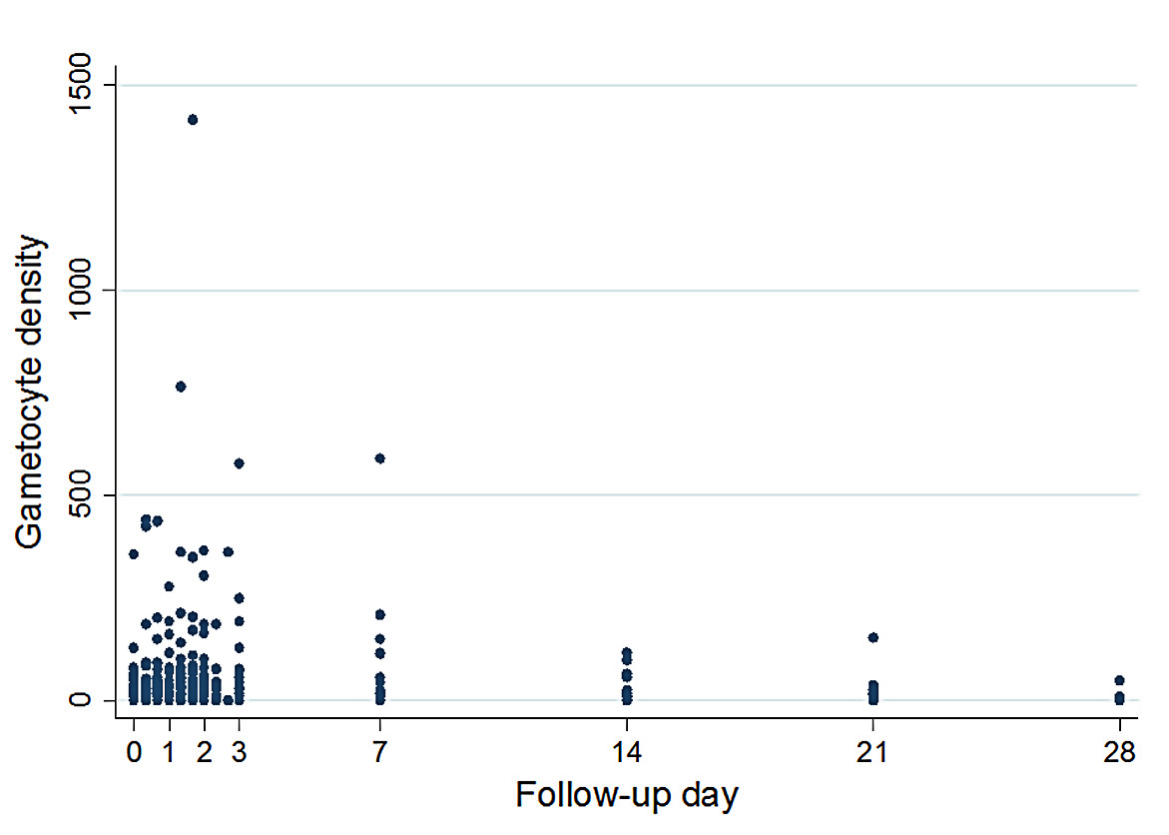
Dynamics of gametocyte density: results are based on all children included from the 2010–2011 study (*n* = 92), not just initial carriers.

**Figure 3.**
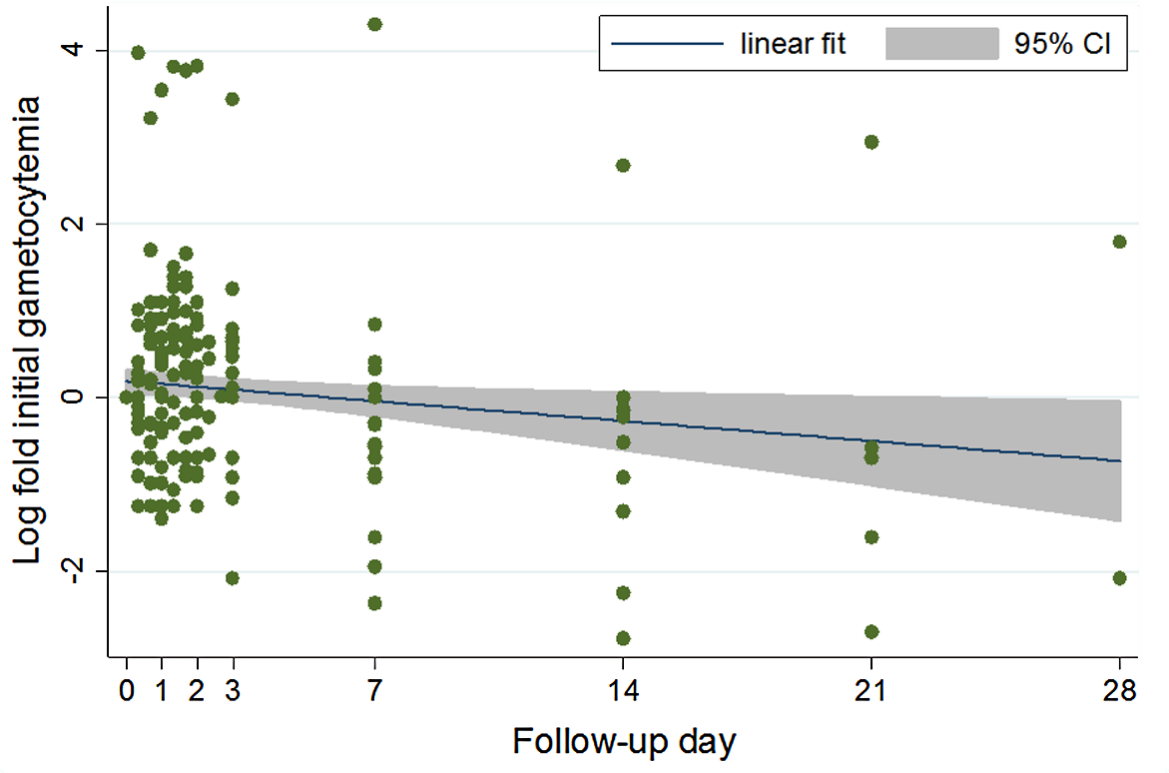
Evolution of gametocyte density among initial carriers: results are based on the 21 children from the 2010–2011 study who were gametocyte carriers at the time of inclusion (H0).

In the 2010–2011 study, the geometric mean gametocyte density at inclusion remained unchanged at the end of treatment (29.3 gametocytes/μL, 95% CI 18.5–46.6 vs. 23.9 gametocytes/μL, 95% CI 13.4–42.5; *p* = 0.6). The number of gametocyte carriers toward the end of the follow-up was too small for any meaningful comparisons (Fig. 2). Figure 3 shows the evolution of gametocyte density among the initial 21 carriers only.

In the 2002–2004 study, the geometric mean gametocyte density at inclusion and day 7 was comparable as well (42.7 gametocytes/μL, 95% CI 25.8–70.5 vs. 73.5 gametocytes/μL, 95% CI 42.1–128.1; *p* = 0.1). There were no gametocyte carriers at day 21 and only 3 carriers at day 28.

Inter-study comparison between 2010–2011 vs. 2002–2004 limiting the analysis to patients recruited during the same months of December and January in each period showed no statistically significant differences in geometric mean gametocyte densities (29.3 gametocytes/μL, 95% CI 18.5–46.6 vs. 42.7 gametocytes/μL, 95% CI 25.8–70.5; *p* = 0.3).

### Gametocyte clearance times

Among gametocyte carriers at inclusion, the median GCT was 336 [72–504] h (interquartile [IQ] range, 72–504 h). In comparison, the median GCT in 2002–2004 was 168 h (IQ range, 48–336 h) (*p* = 0.1). After adjusting for initial gametocyte density, the difference in GCT between the two studies approached but did not reach statistical significance (*p* = 0.09). When the analysis was limited to patients included during the same months in both studies (December–January) the median GCT in 2002–2004 was also found to be 336 h (IQ range 24–336 h) (*p* = 0.4). Note that the GCT could not be calculated for six of the initial gametocyte carriers in the 2010–2011 study because they had either only cleared on the last follow-up day or did not clear at all by the end of the 28-day follow-up period.

### Development of gametocytemia among patients without gametocytemia at baseline

Among patients without gametocytemia at inclusion in the 2010–2011 study, 46.5% (*n* = 71) had gametocytes detected at some point between day 0 and day 7 of follow-up. Of those, 10 (14.1%), 13 (18.3%), 10 (14.1%), and 6 (8.5%) had become carriers by follow-up days 1, 2, 3, and 7, respectively. Similarly, in the 2002–2004 study, among non-gametocyte carriers at inclusion 35.9% (*n* = 64) had gametocytes by day 7 of follow-up. Among those, 7 (10.9%), 10 (15.6%), and 6 (9.4%) became carriers on follow-up days 1, 3, and 7, respectively. Inter-study comparison between 2010–2011 and 2002–2004 limiting the analysis to patients recruited during the same months of December and January in each period showed no statistically significant differences in the proportion of patients with *de novo* gametocytemia (46.5%, *n* = 71 vs. 35.9%, *n* = 64; *p* = 0.2).

## Discussion

We show that in Malian children with uncomplicated malaria, artesunate monotherapy treatment does not clear mature stage V gametocytes from peripheral blood during the 7-day treatment period. In addition, artesunate did not prevent the appearance of new stage V gametocytes in the bloodstream as assessed by light microscopy. To our knowledge, this is the first study to specifically examine the effect of artesunate monotherapy on gametocyte carriage and clearance dynamics in African children with uncomplicated *falciparum* malaria.

Nearly half (46%) of our initial non-gametocyte carriers did develop gametocytemia at some point by day 7 of follow-up, much higher than findings of 9% in a similar study in Vietnam [18]. Two previous studies on African children have found some proportion of immature gametocytes to be unaffected by the addition of artesunate to standard antimalarial regimens, even when gametocytemia is measured submicroscopically [34, 40]. However, although our data indicate that artesunate monotherapy does not kill mature gametocytes, this study cannot rule out the possibility that earlier gametocyte developmental stages may be affected by the drug [16, 17, 22, 25]. Our findings support previous studies demonstrating that the addition of artemisinins to standard antimalarial regimens reduces post-treatment gametocytemia [27, 34, 40] as gametocyte carriage did significantly decrease by the end of the 28-day follow-up. However, these rather late declines could hardly be attributed to artemisinin as its half-life is only a couple of hours [12]. Overall, the data suggest that the decrease in gametocyte carriage observed after ACT treatment may not be due to direct killing of mature or late-stage gametocytes. Rather, it is consistent with the view that artemisinin rapidly and effectively kills asexual stages giving them no time to differentiate into sexual forms. Therefore, it is conceivable that a decrease in efficacy of artemisinins may lead to increased gametocyte carriage in ACT-treated patients, as was demonstrated with SP [5]. Further work is needed to determine the role that sequestered gametocytes may play in the persistence of peripheral gametocytemia after artemisinin-based treatment initiation.

Among the gametocyte carriers at enrollment, our median gametocyte clearance time (GCT) of 336 h in December–January of both 2010–2011 and 2002–2004 is higher than the median GCT of 163 h in a pooled analysis of nine Thai trials of ACTs [30]. This difference may be due to the fact that the Thai data were from various ACT trials, while we used artesunate as monotherapy. Overall, gametocyte clearance in our children population was similar to gametocyte clearance found by Giao et al., who examined gametocyte clearance dynamics in Vietnamese adults treated with a 5- or 7-day course of artemisinin monotherapy [18].

We found a higher proportion of initial gametocyte carriers in the 2010–2011 study than in the entire 2002–2004 study, which may be due to the fact that the latter study was a year-round study while the former was done at the end of the malaria transmission season in this area. Indeed, the proportion of gametocyte carriers was shown to increase throughout the malaria transmission season [15]. However, even when the comparison was limited to the same months, there were significantly more gametocyte carriers at baseline in the 2010–2011 study. The significance of these observations for an evolution in artesunate efficacy requires further larger studies.

An increased duration and density of gametocyte carriage after sulfadoxine-pyrimethamine treatment was proposed as an early indicator of drug resistance [6]. In this study, we found no differences in gametocyte clearance time and no differences in gametocyte densities 6 years after the introduction of artemisinins in this setting. This could be another testimony to the absence of artemisinin resistance in the area at this time [23].

K13 propeller polymorphisms are considered as the best molecular markers of artemisinin resistance [2]. However, the studies establishing these markers were all conducted in South-East Asia [2, 3, 37, 42] where the epidemiology of malaria and most notably antimalarial immunity are much lower than in sub-Saharan Africa. A number of studies have documented low-frequency non-synonymous mutations in the K13 propeller domain of *P. falciparum* isolates from sub-Saharan Africa [19, 24, 41]. However, these SNPs were different from the Asian SNPs and were not associated with any delayed parasite clearance phenotype [29]. In light of the above, monitoring gametocyte carriage, clearance time, and densities may be a useful addition to molecular typing of K13 propeller polymorphisms in the surveillance of artemisinin resistance in sub-Saharan Africa.

The primary limitation of our study is the likelihood of type II bias due to a rather small sample size. Another limitation is our reliance on standard microscopy for gametocyte detection, which is known to consistently underestimate gametocyte carriage [7]. The ability of artesunate to reduce gametocyte prevalence and density in Kenyan children treated with sulfadoxine-pyrimethamine (SP) was more significant when measured by quantitative nucleic acid sequence-based amplification (QT-NASBA) than when measured by microscopy [34]. The GCT is also known to be longer when measured by PCR than by standard microscopy [8]. More sensitive gametocyte detection methods may better characterize gametocyte clearance and carriage dynamics in future work. This is particularly important as models suggest that a submicroscopic gametocyte reservoir may be sufficient to sustain malaria transmission, hampering efforts to eliminate or eradicate malaria [20]. We also did not consider other metrics that may impact infectivity, such as gametocyte sex ratios [36].

Although gametocyte density does not perfectly correlate with infectivity [15], because it is easier to measure it may be a useful indicator of an antimalarial’s probable impact on malaria transmission. Therapeutic efficacy trials of ACTs or individual artemisinins should not neglect to examine the impact of the therapy on gametocyte clearance dynamics following a standardized protocol (same locations, same periods, and same tools). Flow cytometry may be another viable option for assessing the *in vitro* efficacy of artemisinins against gametocyte populations over time to detect early signs of resistance [11].

This study provides baseline gametocyte clearance dynamics when artemisinins are still highly efficacious in sub-Saharan Africa. Monitoring changes in these clearance dynamics may be used as an additional early warning tool in the surveillance of artemisinin resistance in the field.
